# Host barriers to SARS-CoV-2 demonstrated by ferrets in a high-exposure domestic setting

**DOI:** 10.1073/pnas.2025601118

**Published:** 2021-04-15

**Authors:** Kaitlin Sawatzki, Nichola J. Hill, Wendy B. Puryear, Alexa D. Foss, Jonathon J. Stone, Jonathan A. Runstadler

**Affiliations:** ^a^Department of Infectious Disease and Global Health, Cummings School of Veterinary Medicine at Tufts University, North Grafton, MA 01536

**Keywords:** SARS-CoV-2, virology, transmission, genetics, coronavirus

## Abstract

Ferrets (*Mustela putorius furo*) are mustelids of special relevance to laboratory studies of respiratory viruses and have been shown to be susceptible to severe acute respiratory syndrome coronavirus 2 (SARS-CoV-2) infection and onward transmission. Here, we report the results of a natural experiment where 29 ferrets in one home had prolonged, direct contact and constant environmental exposure to two humans with symptomatic disease, one of whom was confirmed positive for SARS-CoV-2. We observed no evidence of SARS-CoV-2 transmission from humans to ferrets based on viral and antibody assays. To better understand this discrepancy in experimental and natural infection in ferrets, we compared SARS-CoV-2 sequences from natural and experimental mustelid infections and identified two surface glycoprotein Spike (S) mutations associated with mustelids. While we found evidence that angiotensin-converting enzyme II provides a weak host barrier, one mutation only seen in ferrets is located in the novel S1/S2 cleavage site and is computationally predicted to decrease furin cleavage efficiency. These data support the idea that host factors interacting with the novel S1/S2 cleavage site may be a barrier in ferret SARS-CoV-2 susceptibility and that domestic ferrets are at low risk of natural infection from currently circulating SARS-CoV-2. We propose two mechanistically grounded hypotheses for mustelid host adaptation of SARS-CoV-2, with possible effects that require additional investigation.

## Significance

Ferrets have been demonstrated to be susceptible to laboratory infection of SARS-CoV-2, raising the possibility of natural transmission from humans into their pets in domestic settings. We demonstrate that ferrets may have host barriers that limit natural infection and transmission. First, we find no evidence of infection in 29 ferrets from a home with constant exposure to two adults with one confirmed and one suspected case of symptomatic COVID-19. Second, we analyze genetic sequences from viruses and hosts and demonstrate that ferrets have genetic factors that confer resistance to natural SARS-CoV-2 infection. These data suggest that ferret infection may require viral adaptation, and therefore ferrets may only be semipermissive models of SARS-CoV-2 disease or transmission.

Severe acute respiratory syndrome coronavirus 2 (SARS-CoV-2), the virus that causes COVID-19, is a zoonotic member of *Coronaviridae* that emerged in 2019 as a major viral pandemic (1). As of February 2021, there have been ∼102 million confirmed COVID-19 cases globally and ∼2.2 million deaths (2). SARS-CoV-2 uses angiotensin I converting enzyme-2 (ACE2) as its primary cellular receptor for host entry and infection (3–5). In silico analyses of ACE2 genes in diverse mammalian species show that residues important to viral binding are moderately conserved between humans and several domestic animals, and a broad range of species have been demonstrated to be permissive to infection in vitro and in vivo (6–10).

It is not yet known whether natural infection of animals plays a role in public health epidemiology or has the potential to establish endemic reservoirs and threaten wildlife. SARS-CoV-2 has been observed to be capable of natural human-to-animal reverse zoonoses, transmitting from infected individuals into mink (11), dogs (12), and felines (13–15). American mink (*Neovison vison*) are currently the only species observed to have natural human-to-animal spillover and onward transmission (11). To date, at least 27 mink farms in The Netherlands, Spain, Denmark, and United States have reported outbreaks, including at least one probable case of mink-to-human transmission (16, 17).

SARS-CoV-2 has also been shown to productively infect several species, including ferrets and domestic cats, in vivo (9, 10, 18, 19). Ferrets (*Mustela putorius furo*) are of special relevance to laboratory studies of respiratory viruses like *Influenza A virus* and recapitulate clinical pathophysiological aspects of human disease. Given their susceptibility to experimental infection and onward transmission via direct and indirect contact, ferrets have been proposed as an animal model to study SARS-CoV-2 transmission. Based on in vivo data, we expect all naïve ferrets in direct contact with an infected ferret will 1) become infected, 2) have measurable viral shedding or RNA via oral swabs up to 19 d postinfection, and 3) seroconvert with measurable antibodies against SARS-CoV-2 receptor binding domain (RBD) (18, 19).

In March 2020, during the first wave of the SARS-CoV-2/COVID-19 pandemic in the New England area, we developed a rapid response study to investigate the potential for human-to-animal spillover and onward transmission in domestic, farm, and wildlife species (CoVERS: Coronavirus Epidemiological Response and Surveillance). The goal of CoVERS is to understand whether and how SARS-CoV-2 transmission is occurring at these interfaces, to refine public health guidelines, investigate whether there are additional risks to animal or human health associated with spillover, and evaluate the potential for establishment of endemic reservoirs. In the CoVERS in-home study, participants are sent a “swab and send” kit, which provides materials and instructions to safely take longitudinal nasal and oral samples from their animals, store them in their freezers, and send them back for viral screening. This community science approach allows wide surveillance with no risk of human transmission, as kits are decontaminated and opened in biosafety cabinets. Here, we highlight one enrolled household that created an exceptional natural experiment with direct relevance to our understanding of SARS-CoV-2 reverse zoonosis and animal models of disease.

## Results

### Absence of Natural SARS-CoV-2 Human-to-Ferret Transmission in a High-Exposure Setting.

**Fig. 1.**
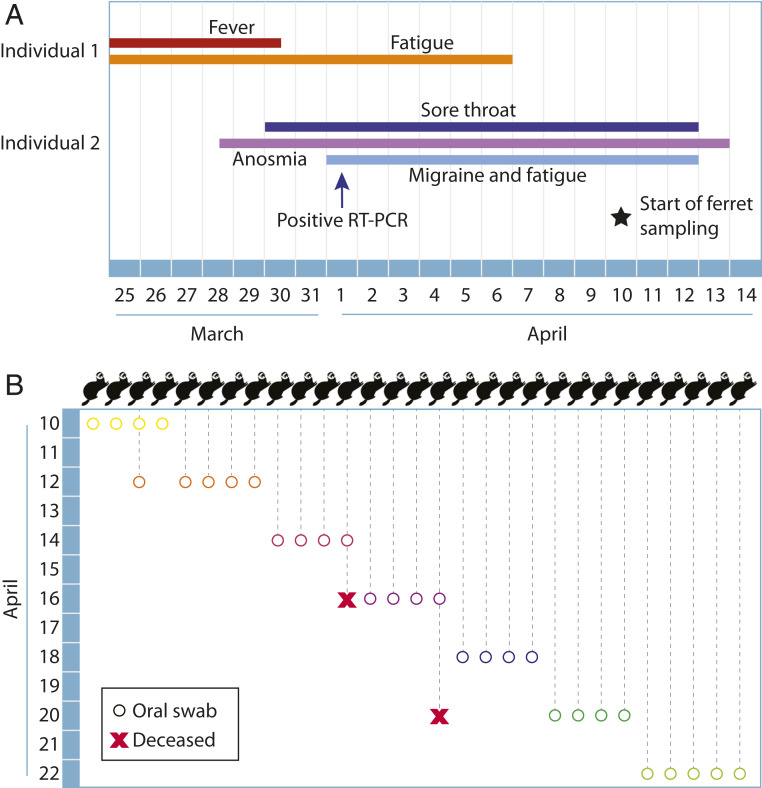
COVID-19 disease course and ferret sample collection timeline. A household with two adults and 29 free-roaming ferrets was enrolled in the CoVERS study. (*A*) Both adults exhibited symptoms of SARS-CoV-2 infection in late March to early April of 2020, and one tested positive by RT-PCR on 1 April. (*B*) Oral swabs were collected from all ferrets in the home over a 2-wk period, beginning April 10, concurrent with symptomatic disease in Individual 2. One ferret (subject 3) was sampled twice. Two 7-y-old ferrets (subjects 12 and 16) died during the study period, one by euthanasia due to chronic disease, the other cause is unknown.

A household with 29 free-roaming ferrets cared for by two related, adult housemates was enrolled in the CoVERS study. Individual 1 experienced fever and fatigue from March 25 to April 6, and individual 2 experienced a sore throat, anosmia, migraine, and fatigue from March 28 to April 13 (Fig. 1*A*). Individual 2 tested positive for SARS-CoV-2/COVID-19 infection by nasopharyngeal swab and RT-PCR on April 1. Individual 1 is a suspected positive due to the timing and symptoms but was not tested. Neither person was hospitalized, and both cared for the ferrets during the entirety of their disease courses.

A 2-wk, in-home sample collection scheme was designed to begin during the household quarantine period (Fig. 1*B*). The ferrets were free to move in all spaces of the home during this period and handled as usual, including daily petting, feeding, and grooming. Individual 1 described daily close contact with holding, laying, and/or cuddling with all ferrets, as well as daily cage cleaning. The ferrets ranged in age from 8 mo to 7.5 y over 21 females and 8 males. A home sampling kit was sent to the participants, including material to safely collect and store ferret oral swabs. One participant had significant animal handling experience and performed all sample collection to standardize sampling procedures. Thirty oral swabs were collected and held in viral transport media in the participants’ freezer until the end of the study period. Frozen samples were directly transferred to a laboratory member and processed.

All samples were confirmed to have viable RNA, by a preliminary screen for constitutively expressed β-actin (Table 1). Each sample was then tested for evidence of active or recent SARS-CoV-2 infection, with three established primer sets: Open Reading Frame 1b nonstructural protein 14 (ORF1b) (20), Nucleocapsid (N) (14), and RNA-dependent RNA polymerase (RdRP) (21). All were below the limit of detection and determined to be negative for active or recent infection (Table 1).

**Table 1.** No evidence of SARS-CoV-2 infection in ferrets

Ferret	ACTB	ORF1b	N	RdRP	Total IgG	αRBD IgG
1	33.036	LOD	LOD	LOD	P	N
2	28.120	LOD	LOD	LOD	P	N
3a	27.954	LOD	LOD	LOD	P	N
3b	28.945	LOD	LOD	LOD	P	N
4	26.230	LOD	LOD	LOD	P	N
5	29.067	LOD	LOD	LOD	P	N
6	29.729	LOD	LOD	LOD	P	N
7	29.360	LOD	LOD	LOD	P	N
8	26.755	LOD	LOD	LOD	P	N
9	33.049	LOD	LOD	LOD	P	N
10	32.820	LOD	LOD	LOD	N	NA
11	29.781	LOD	LOD	LOD	P	N
12	29.010	LOD	LOD	LOD	P	N
13	27.730	LOD	LOD	LOD	N	NA
14	32.163	LOD	LOD	LOD	P	N
15	30.230	LOD	LOD	LOD	P	N
16	27.861	LOD	LOD	LOD	P	N
17	27.701	LOD	LOD	LOD	P	N
18	27.687	LOD	LOD	LOD	N	NA
19	30.832	LOD	LOD	LOD	N	NA
20	31.758	LOD	LOD	LOD	P	N
21	31.758	LOD	LOD	LOD	N	NA
22	32.635	LOD	LOD	LOD	P	N
23	27.098	LOD	LOD	LOD	P	N
24	29.290	LOD	LOD	LOD	P	N
25	29.806	LOD	LOD	LOD	N	NA
26	35.042	LOD	LOD	LOD	N	NA
27	30.032	LOD	LOD	LOD	P	N
28	31.464	LOD	LOD	LOD	P	N
29	29.476	LOD	LOD	LOD	P	N

Ferret oral swabs were tested by semiquantitative real time RT-PCR and ELISA. Sample and RNA viability was confirmed by β-actin (ACTB). Three separate primers sets were used to test for SARS-CoV-2: ORF1b, N, and RdRP; LOD denotes under the limit of detection. ELISA was performed twice for total IgG antibodies against recombinant protein A/G (Total IgG) and purified SARS-CoV-2 RBD (αRBD IgG). ELISA results presented are negative (N), positive (P), or not applicable (NA) if there is insufficient total IgG,

We further took advantage of salivary immunoglobulin G (IgG), which has been shown to be highly sensitive and specific for SARS-CoV-2 testing (22). We tested samples for evidence of antibodies against SARS-CoV-2 surface glycoprotein RBD. Twenty-two ferrets (23 total samples) were confirmed to have measurable total IgG via binding to recombinant protein A/G but were all negative for binding to RBD (Table 1). Therefore, there is no evidence of viral infection or seroconversion in 29 ferrets living with two people with COVID-19.

### Identification of Two Mustelid-Associated Mutations in SARS-CoV-2 Surface Glycoprotein.

**Fig. 2.**
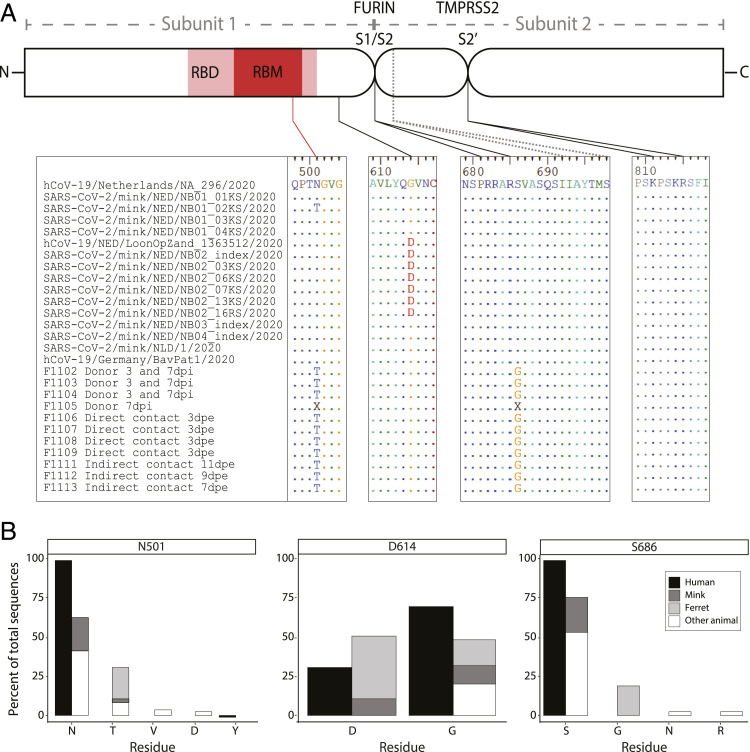
Mustelid-associated mutations in SARS-CoV-2 surface glycoprotein. SARS-CoV-2 surface glycoprotein (S) sequences from natural (mink) and experimental (ferret) infections were compared, and three mutations were identified. (*A*) A schematic diagram (not to scale) of the S protein with Subunit 1, which is involved in host receptor protein attachment, and Subunit 2, which is involved in host cell fusion. Mutation N501T is located in the RBD and receptor binding motif (RBM), shown in red. Mutation D614G is located in Subunit 1 downstream of the RBD, and mutation S686G is located directly adjacent to the novel S1/S2 cleavage motif (PPAR↓**S**) processed by furin. A second S1/S2 cleavage site (IAY↓TMS) seen in SARS-CoV is conserved. The S2′ cleavage site (KPSKR↓S) processed by TMPRSS2 is also conserved. Viral amino acid sequences from regions of interest are shown below the schematic, and dots represent conserved residues, using the top sequence as a reference (hCoV-19/Netherlands/NA_296/2020). Viruses from mink are separated into two clades from distinct farms (NB01 and NB02-4, respectively), and are preceded by the closest observed human sequence (hCoV-19/Netherlands) for reference. Experimentally infected ferrets are in the bottom half (F1102 to F1113). The sequence from the human inoculum (hCoV-19/Germany) is included for reference. Ferrets are separated into three groups: donors, which received direct inoculum; direct contact, which were housed with donors; and indirect contact, which were housed adjacent to donors without physical contact. Identical sequences were found from samples taken at 3 and 7 d post inoculation (dpi) in three of four donors. Donor F1105 exhibited two equivalent single-nucleotide variants (A1502C and A2056G) resulting in N501/N501T and S686/S686G, respectively, and not consensus called (“X”) in those locations. (*B*) The 9,253 human-derived SARS-CoV-2 S protein sequences and 57 animal-derived SARS-CoV-2 or SARS-CoV−like virus S protein sequences were aligned to calculate percent amino acid representation at three positions: N501 (top), D614 (middle), and S686 (bottom).

Our observed household data support the idea that there may be important barriers to natural infection in ferrets; however, ferrets have been shown to be susceptible to infection and onward transmission in experimental laboratory infections (9, 10, 18, 19). To further investigate this, we analyzed available genomic sequences of SARS-CoV-2 viruses of naturally infected American minks and experimentally infected ferrets (32 sequences representing 24 animals, accessed 1 August 2020). There are viral sequences available from two natural reverse zoonotic events in mink farms in Europe, which allowed us to infer founder-effect mutations versus acquired mutations of relevance to spillover (11). We identified three mutations of interest in the surface glycoprotein (S protein) coding sequence: N501T, D614G, and S686G (Fig. 2*A*).

First, N501T was observed in 11/11 experimentally infected ferrets (donor, direct, and indirect contacts), with an increasing proportion of the virome represented through the study period, supporting strong positive selection in ferrets (19). Only 1 of 13 mink viruses is N501T, which supports spontaneous mutation and natural selection in the population. The measured mutation rate calculated from the closest observed human-derived sequences in mink is very low, 4.2 × 10^−4^ substitutions per site, so we asked whether this specific mutation is otherwise common and not unique to mustelid infection. Of 9,049 high-quality human-derived SARS-CoV-2 S genes, none exhibit the N501T mutation (Fig. 2*B*). However, N501T is seen in 5/17 pangolin-derived SARS-CoV-2−like viruses. Notably, the equivalent residue in SARS-CoV, which caused an outbreak in 2003, is a threonine (T487), further supporting that this may be a functionally relevant site.

We observed a second conserved mutation, D614G, in one of the two mink clades and all ferrets. However, this mutation has become prevalent in the human population (D614, 30.5%; D614G, 69.5%; Fig. 2*B*) and was observed in the ferret human donor and mink farm’s closest observed ancestor (Fig. 2*A*). We infer that D614G mutations are due to variation in the human population/donors and are not specifically associated with mustelid infection.

The third nonsynonymous S protein mutation, S686G, was only observed in ferrets and is located at the P1′ serine residue directly adjacent to the novel S1/S2 polybasic cleavage site (PRRAR↓**S**) (Fig. 2*A*). This mutation is of special interest, as this cleavage site partially distinguishes SARS-CoV-2 from other SARS-like viruses and allows immune evasion prior to receptor binding (23–25). Like N501T, S686G was observed in 11/11 ferrets, was a minority variant in the donor inoculum, and increased proportional representation in the virome over time, suggesting positive selection (19). We found that no other human-derived viral sequence has been observed with this mutation (Fig. 2*B*). S686G has also not been observed in SARS-CoV-2−like viruses from other *Carnivora* (naturally infected felines and canines), all of which retained the complete cleavage site and adjacent P1′ serine. All mustelid-derived viruses retained the second, downstream S1/S2 cleavage site motif (IAY↓TMS), as well as the S2′ TMPRSS2-processed cleavage site for fusion.

Host furin and furin-like proteases have been shown to cleave the S1/S2 polybasic cleavage site (3, 25, 26). P1′ residues are strongly favored to be serine in furin cleavage, and alternate residues are restricted by size and hydrophilicity due to their location in the furin binding pocket (27). Glycine is small but hydrophobic. We performed in silico analysis of the cleavage site to compare identical sequences that differed only at position 686, using PiTou 2.0 (28). PiTou scores are biologically meaningful prediction values of furin cleavage derived from binding strength and solvent accessibility. S686 results in a PiTou score of 9.19633, while S686G results in a score of 6.92387. While both are predicted to be cleaved by furin, S686 is estimated to have stronger interactions in the binding pocket (P6 to P2′). Therefore, S686G is an unfavorable substitution for furin cleavage.

We further performed phylogenetic analysis of the proprotein convertase family that cleaves polybasic sites (PCSK1 to 7), including furin, and Cathepsin L in a number of mammals, including ferret and the well-annotated ermine. However, we found no clear differences between ferrets, ermines, and other members of *Carnivora* (*SI Appendix*, Figs. S1–S8).

## Discussion

Multiple studies have now demonstrated that ferrets may be directly infected by human-derived SARS-CoV-2 and, following infection, exhibit a nearly 100% transmission rate via direct contact (9, 10, 18, 19, 29). Recent reports also describe sporadic cases of natural infection (30, 31). However, our data suggest that the initial barrier of human-to-ferret transmission may be higher than relevant to most household pets. We calculated that a sample size of 10 animals was sufficient to test the hypothesis that at least one ferret was infected, given an observed attack rate of 87% in mink farms (95% CI, 0.05) (32). In this natural experiment, all 29 ferrets had significant opportunities for direct contact with all other ferrets and had direct exposure to at least one infected person.

Based on current knowledge of SARS-CoV-2 transmission and shedding in ferrets, we determined that our collection time points fell within the timeframe to obtain measurable viral RNA, even if transmission occurred on March 22, prior to any symptom onset in the household. However, it was important to perform additional antibody testing to address two concerns: first, that transmission could have occurred prior to March 22 and, second, that the level of infection and viral shedding was so low as to be below collection and screening sensitivity. In either scenario, we still expected a robust antibody presence within days of initial infection but found no evidence of RBD-specific antibodies. Despite significant and prolonged exposure in the home, we have concluded that there is no evidence of SARS-CoV-2/COVID-19 human-to-ferret transmission in this household.

An important caveat of this transmission study is the question of infectiousness in humans. We were unable to collect human samples in this work, and therefore we suspect, but cannot prove, that both adults had an infectious period. Symptomatic infection is correlated with contagiousness, and both cases had moderate symptomatic disease (Fig. 1*A*) (33). Based on symptom onset, we suspect that individual 1 may have been infectious while in the home and transmitted SARS-CoV-2 to individual 2 (34, 35).

Notably, ferret 12 (7 y old) was euthanized on April 16, and had a history of adrenal disease, and ferret 16 (7 y old) died unexpectedly on April 20. Both were swabbed within 4 d of their deaths and, we expect, would have been RT-PCR or antibody positive had their deaths been related to SARS-CoV-2 infection.

We found no evidence of SARS-CoV-2 transmission to ferrets, a finding at odds with the high transmission rates observed in laboratory and mink farm settings. Further evidence of natural transmission to household ferrets has exhibited a lower prevalence than those seen in dogs and cats, with only one currently confirmed case worldwide (15, 30, 31). To investigate whether viral or host genetics played a role in this discrepancy, we utilized available data for analysis and biologically relevant hypothesis generation using computational tools. Viral host receptors are often a key factor in determining host range. American minks and ferrets share 24 of 25 ACE2 residues with known viral S protein interactions, and we expect these species to have similar natural susceptibility (7). N501T is in the receptor binding motif of the SARS-CoV-2 surface glycoprotein, which interacts with ACE2 primarily at Y41, but also K353, G354, and D355 (36, 37). Of these, mustelids only differ from humans at ACE2 G354, and this site is also the only distinct residue between ferret (G354R) and American mink (G354H) (7). Mink have been naturally infected by virus without the N501T mutation, and there have now been dozens of independent human-to-mink spillover events; therefore, we do not expect that the ACE2 G354H mutation significantly limits infection. However, the appearance of N501T in all infected ferrets suggests ACE2 G354R may provide a host barrier to SARS-CoV-2 entry in ferrets. N501T has appeared as a spontaneous mutation in four mink from four independent farm outbreaks (38). Additional work is needed to determine whether N501T is a required adaptation for ferret transmission and, if so, how it affects transmission dynamics. The increasing prevalence of N501Y among human populations has further raised concerns of increased transmissibility, virulence, and immune evasion related to mutations at this position (39, 40).

SARS-CoV-2 S protein S686G is another intriguing mutation, as it lies directly adjacent to a motif that is likely to enhance virulence (25). As of sequence accession on July 15, 2020, S686 is perfectly conserved in 9,189/9,189 human sequences, indicating strong purifying selection. S686G changes a neutral polar residue to a nonpolar one, which we estimated to decrease furin efficiency. Furthermore, S686 completes a novel glycosaminoglycan (GAG)-binding motif (XBBXBX/PRRARS) that enhances binding, and the two flanking serines in the S1/S2 site (SPRRAR↓SV) have been shown to be permissive to host phosphorylation and consequent down-regulation of furin activity (26, 41). For these reasons, we were surprised to see evidence of positive selection over time for this potentially unfavorable mutation in ferrets as described by Richard et al. (19). If there is further evidence of S686G selection in experimentally or naturally infected ferrets, it is essential to fully investigate changes in viral fusion activity, kinetics, and pathology to determine whether ferrets are an appropriate model for human disease.

Beginning with our initial community science-based surveillance efforts, we observed an unexpected result and went on to use publicly available data to investigate biologically relevant mutations correlated to species-specific infection in an important laboratory model. In addition to providing data to better evaluate risk to pet ferrets, this study unexpectedly led to broader hypotheses about mustelid susceptibility and resistance to SARS-CoV-2 with implications for experimental research and wildlife disease ecology. We propose that the mustelid-specific viral mutations we have identified have biological relevance to infection efficiency and transmission. In laboratory models, positive selection for rare variants away from human wild-type virus may affect outcomes and should be further investigated. Recent campaigns to vaccinate the endangered black-footed ferret and farmed mink are also important for continued, targeted investigation, as our results may mean that inoculation with a nonmustelid adapted variant may not provide sufficient protection.

Our results suggest that virus and host genetic barriers significantly limit natural infection in ferrets, and these are only likely to be overcome by a concentrated and/or diverse inoculum of human-derived virus. To date, successful experimental ferret infections have used 3 × 10^5^ to 6 × 10^5^ 50% tissue culture infectious dose (TCID_50_) virus, and at least one inoculum contained a minority of virus with the N501T and S686G variants (18, 19). These limitations and putative host adaptations may negatively affect ferrets as a disease or transmission model and should be further investigated. The data presented here remind us that synthesis of data from surveillance work, natural experiments, and controlled, laboratory-led studies can lead us to novel hypotheses and investigations and allow us to better respond to this pandemic and prepare for the next. This household provides evidence that human-to-ferret SARS-CoV-2 transmission in domestic settings may be lower risk than would be expected from laboratory experiments.

## Materials and Methods

### Study Enrollment and Sample Collection.

Study participants were enrolled under a protocol and consent form approved by Tufts University Institutional and Animal Care and Use Committee and Health Sciences Institutional Review Board (#G2020-27) and provided signed consent to take a voluntary questionnaire and for animal specimens to be self-collected and tested for SARS-CoV-2 and Influenza A virus. A self-administered sampling kit was sent to the enrollees’ residence with sterile standard polyester-tipped applicators (Puritan), vials with 800 μL of M4RT viral transport media (Remel), instructions, a data sheet, and secondary containment bags. Oral swabs were obtained using gloves and a mask in the home and held in a home freezer until transfer to a laboratory member via a cooler.

### RNA Extraction and RT-PCR.

Samples were vortexed, and 50 μL was aliquoted for MagPlate OMEGA extraction following manufacturer protocols. RNA was tested by semiquantitative real time RT-PCR on the StepOnePlus platform (ABI) with qScript XLT 1-Step RT-PCR ToughMix, using five primer sets: one for internal controls (ACTB) and three for SARS-CoV-2 (ORF1b, N1, RdRP). CoVERS-ACTB, Forward: GAT​GCA​GAA​GGA​GAT​CAC, Reverse: CTA​GAA​GCA​TTT​GCG​GTG, Probe: HEX-CTCCTGCTTGCTGATCCACA-TAM; HKU-ORF1, Forward: TGGGGYTTTACRGGTAACCT, Reverse: AACRCGCTTAACAAAGCACTC, Probe: FAM-TAGTTGTGATGCWATCATGACTAG-TAM; 2019-nCoV_N1 [CDC], Forward: GAC​CCC​AAA​ATC​AGC​GAA​T, Reverse: TCT​GGT​ACT​GCA​GTT​GAA​TCT​G, Probe: FAM-ACCCCGCATTACGTTTGGTGGACC-TAM; RdRP_SARSr, Forward: GTGARATGGTCATGTGTGGCmGG, Reverse: CARATGTTAAASACACTATTAGCAmTA, Probe: FAM-CAGGTGGAACCTCATCAGGAGATGC-TAM. All plates were run with negative viral transport medium (VTM) controls and positive control (NR-52285, Genomic RNA from SARS-Related Coronavirus 2, Isolate USA-WA1/2020, BEI Resources).

### ELISA.

Oral swabs were tested for total IgG and IgG against SARS-CoV-2 RBD with minor modifications to an established protocol (42). Briefly, Immulon 2 HB plates were coated with 2 μg/mL Pierce recombinant protein A/G (ThermoFisher catalog no. 77677) or purified SARS-CoV-2 RBD (provided by Florian Krammer, Icahn School of Medicine at Mount Sinai, New York, NY; available as NR-52366, BEI Resources) and incubated for 2 d at 4 °C. After washing, plates were blocked with phosphate-buffered saline (PBS) supplemented with 0.1% Tween-20 (PBS-T) and 3% milk at room temperature for 2 h. All samples were heat inactivated at 56 °C for 1 h. Ferret samples were diluted 1:5 in PBS-T with 1% milk. Positive controls were serum from S protein-immunized alpacas (provided by Charles Shoemaker, Cummings School of Veterinary Medicine at Tufts University, North Grafton, MA) and diluted 1:5 in PBS, then to a final dilution of 1:50 in PBS-T with 1% milk. Following blocking, 100 μL of diluted samples were incubated at room temperature for 2 h. Plates were washed, and 50 μL of Pierce recombinant protein A/G with peroxidase (Thermo Fisher catalog no. 32490) was added at 1:10,000 in PBS-T with 1% milk and incubated for 1 h at room temperature. Plates were washed and developed for 10 min with SigmaFast o-Phenylenediamine dihydrochloride solution (Sigma-Aldrich catalog no. P9187), stopped with 50 uL of 3M HCl, and read at an absorbance of 490 nm on a BioTek Synergy 4 Multidetection plate reader. Positive cutoff was set at (μ + 3σ) of the negative controls (*n* = 24). VTM was tested at 1:2 and 1:5 dilutions and confirmed to not affect results.

### Viral Sequence Collection and Assembly.

High-quality SARS-CoV-2 surface glycoprotein sequences were curated using National Center for Biotechnology Information (NCBI) Virus and Global Initiative on Sharing All Influenza Data (GISAID) EpiCoV databases as follows (43, 44): 9,272 full-length S nucleotide sequences were collected from NCBI Virus and aligned using ClustalΩ(v1.2.4) (45). Sequences were trimmed to coding region sequence, translated, and realigned. Sequences with >10% unknown residues were excluded. Twenty-seven additional nonhuman animal-derived SARS-CoV-2 and SARS-CoV-2−like viral sequences were collected from GISAID EpiCoV. GISAID attributions are in Supplemental Dataset 1. To collect viral genomes from experimental ferret infection, sequencing reads were downloaded from 23 Illumina and Minion sequencing runs uploaded to NCBI Sequence Read Archive (PRJNA641813). Reads were confirmed to be post quality control by Prinseq and mapped to the human donor sequence (hCoV-19/Germany/BavPat1/2020|EPI_ISL_406862|2020-01-28) using Burrows-Wheeler Aligner (BWA) for Illumina data and Pomoxis mini_align for Minion data (46, 47). Consensus was called using SAMtools, and replicate Illumina/Minion libraries were compared to confirm consistency (48). Aligned sequences are available on a GitHub repository (49).

### Mammalian Gene Collection, Assembly, and Phylogenetic Analysis.

PCSK1-7 and CTSL sequences were collected from NCBI Orthologs from *Homo sapiens* (human), *Pan troglodytes* (chimpanzee), *Sus scrofa* (pig), *Ovis aries* (sheep), *Bos Taurus* (cow), *Canis lupus familiaris* (dog), *Canis lupus dingo* (dingo), *Vulpes vulpes* (fox), *Felis catus* (cat), *Panthera tigris altaica* (Siberian tiger), *Lontra canadensis* (river otter), *Enhydra lutris* (sea otter), *Phoca vitulina* (harbor seal), *Mustela erminea* (ermine), *Myotis lucifugus* (little brown bat), *Eptesicus fuscus* (big brown bat), *Rousettus aegyptiacus* (Egyptian fruit bat), *Rhinolophus ferrumequinum* (greater horseshoe bat), and *Pteropus vampyrus* (large flying fox). *M. p. furo* (ferret) orthologs were inconsistent with related species by preliminary RAxML ortholog analysis (50). Seven available RNAseq run from *M. p. furo* (SRR11517721-SRR11517724, SRR391982, SRR391968, SRR391966) were downloaded, and putative PCSK1-7/CTSL reads were extracted using Basic Local Alignment Search Tool (BLAST) (51). Reads were assembled using Pomoxis mini_assemble with ermine references. Reads were then mapped back to the proposed ferret assembly with BWA, and well-supported consensus sequences were called using SAMtools. Ortholog collections were analyzed using maximum likelihood phylogenetics via RAxML (JTTγ using empirical base frequencies, 5,000 bootstraps) (50). RAxML output are available on a GitHub repository (49).

## Supplementary Material

Supplementary File

Supplementary File

## Data Availability

Alignment and phylogenetic data from genetic sequences have been deposited in a GitHub repository, https://github.com/ksawatzki/Mustelid_COVID_PNAS (49). All other data are included in the manuscript and/or supporting information.
