# Supplementary material for: Host barriers to SARS-CoV-2 demonstrated by ferrets in a high-exposure domestic setting
Source: Proc Natl Acad Sci U S A. 2021 Apr 15;118(18):e2025601118. doi: 10.1073/pnas.2025601118 (PMC8106344; doi:10.1073/pnas.2025601118)
Supplement: Supplementary File [file pnas.2025601118.sapp.pdf]

Supplementary Information for
Host barriers to SARS-CoV-2 demonstrated by ferrets in a high-exposure domestic setting

Kaitlin Sawatzki, Nichola J. Hill, Wendy B. Puryear, Alexa D. Foss, Jonathon J. Stone and Jonathan A. Runstadler

Corresponding author: Kaitlin Sawatzki
Email: kaitlin.sawatzki@tufts.edu

This PDF file includes:

Figures S1 to S8

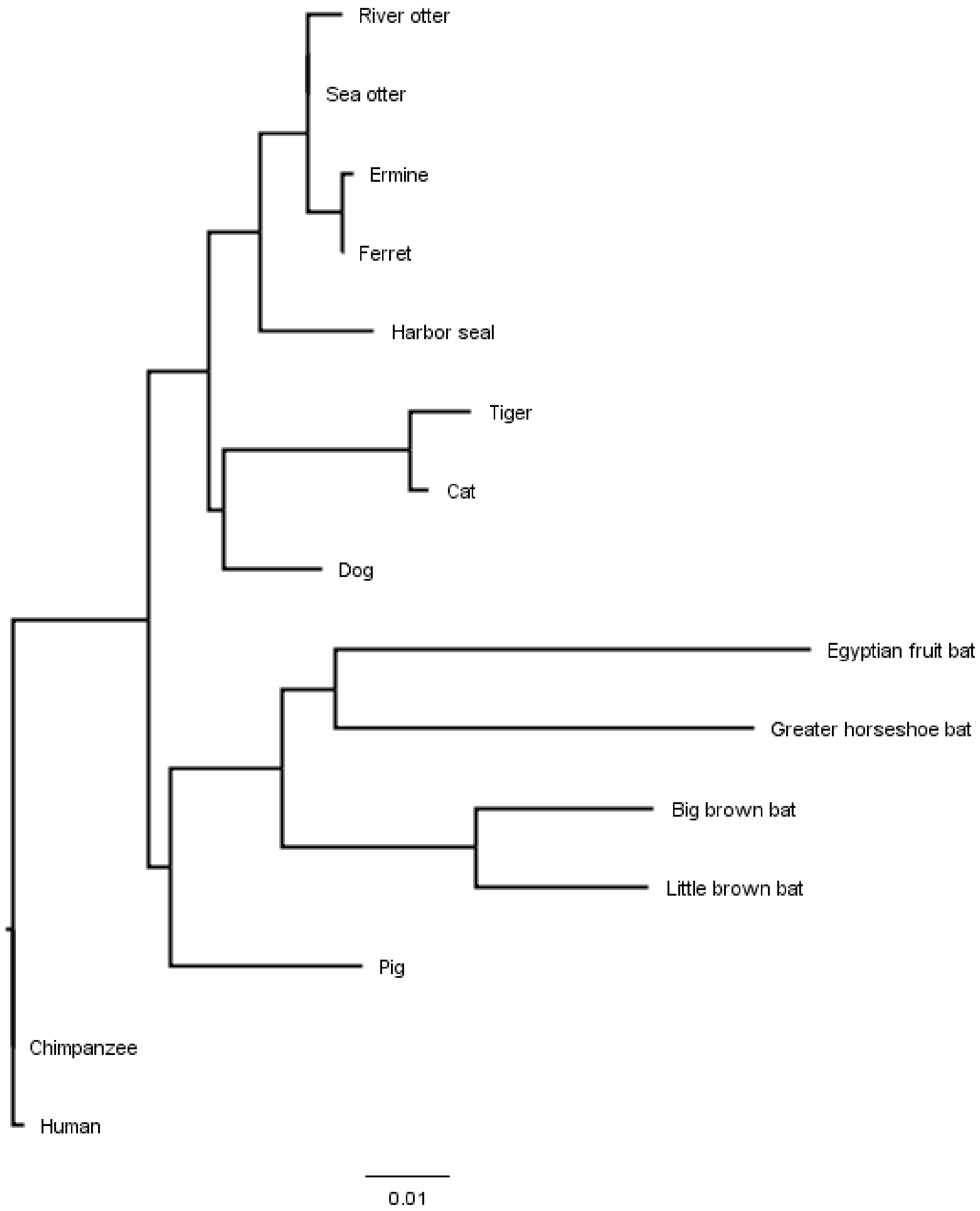

Fig. S1. Maximum likelihood tree of Proprotein Convertase Subtilisin/Kexin Type 1 (PCSK1) among representative mammals. Orthologs were aligned as amino acid sequences (n=15) and a tree generated using RAxML under the JTTγ model with bootstrapping (x=5000) to evaluate reliability of tree topology. Shown in increasing node order. Scale bar corresponds to mutation rate.

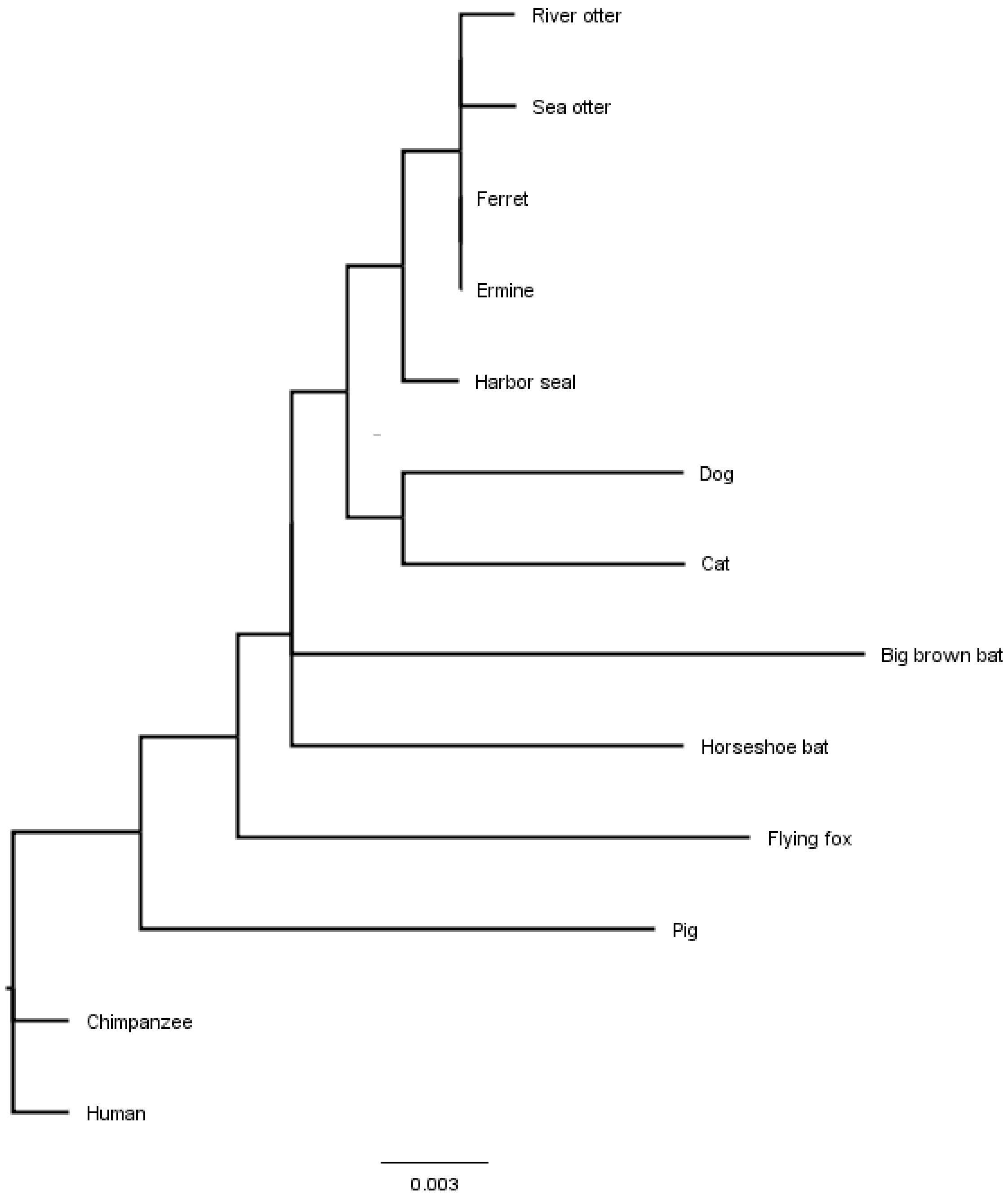

Fig. S2. Maximum likelihood tree of Proprotein Convertase Subtilisin/Kexin Type 2 (PCSK2) among representative mammals. Orthologs were aligned as amino acid sequences (n=13) and a tree generated using RAxML under the JTTγ model with bootstrapping (x=5000) to evaluate reliability of tree topology. Shown in increasing node order. Scale bar corresponds to mutation rate.

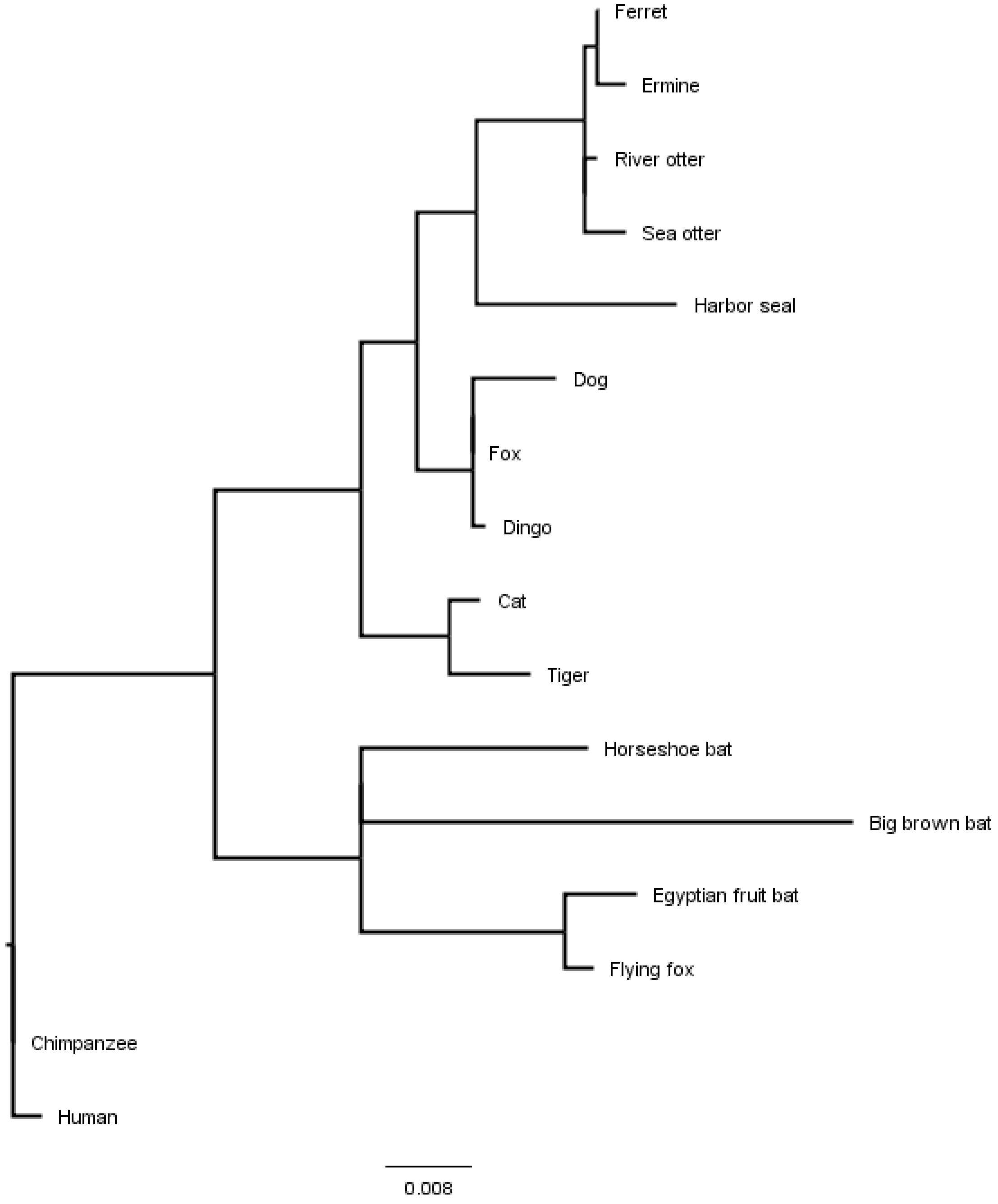

Fig. S3. Maximum likelihood tree of Furin (FUR, PCSK3, PACE) among representative mammals. Orthologs were aligned as amino acid sequences (n=16) and a tree generated using RAxML under the JTTγ model with bootstrapping (x=5000) to evaluate reliability of tree topology. Shown in increasing node order. Scale bar corresponds to mutation rate.

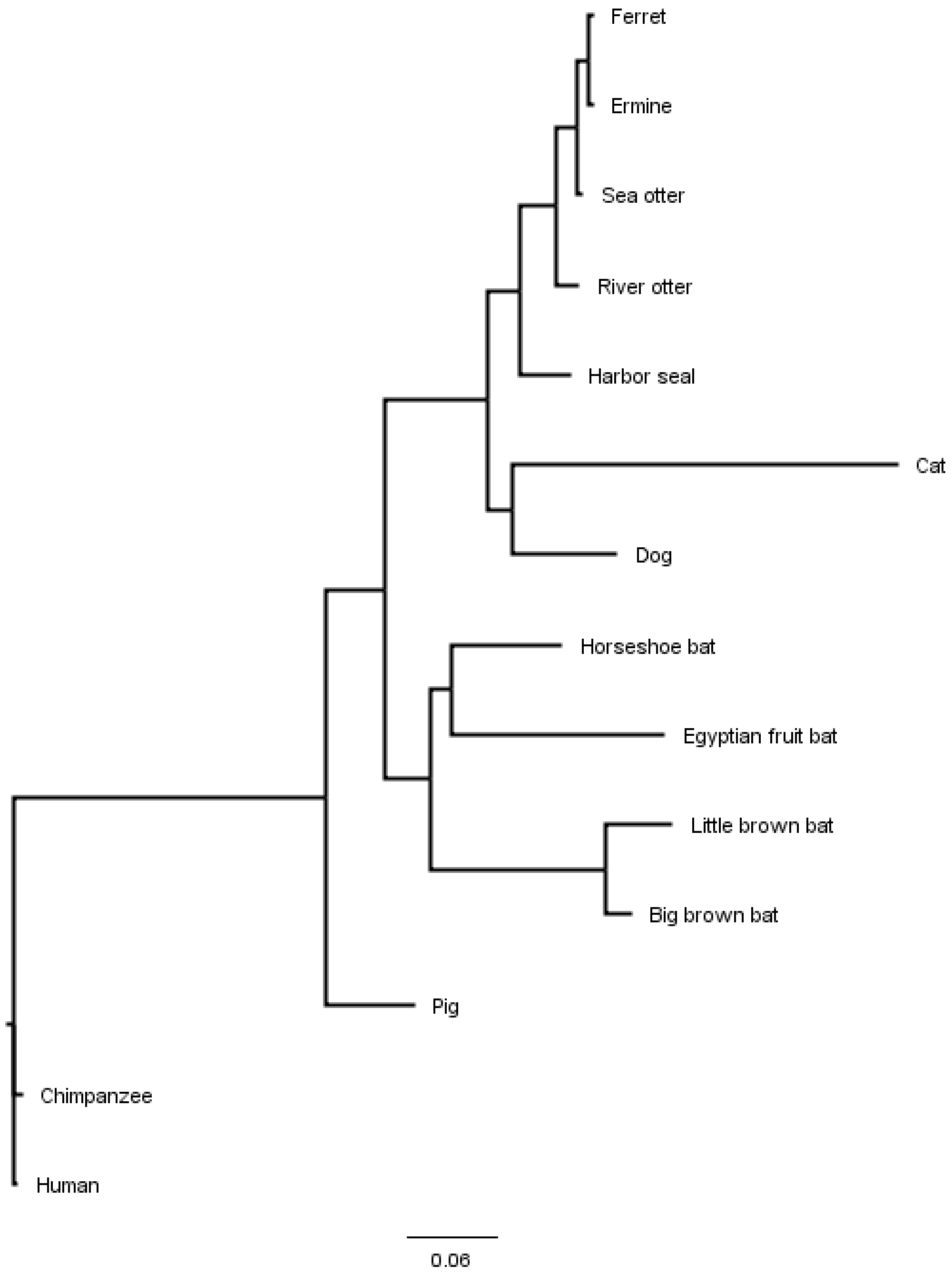

Fig. S4. Maximum likelihood tree of Proprotein Convertase Subtilisin/Kexin Type 4 (PCSK4) among representative mammals. Orthologs were aligned as amino acid sequences (n=14) and a tree generated using RAxML under the JTTγ model with bootstrapping (x=5000) to evaluate reliability of tree topology. Shown in increasing node order. Scale bar corresponds to mutation rate.

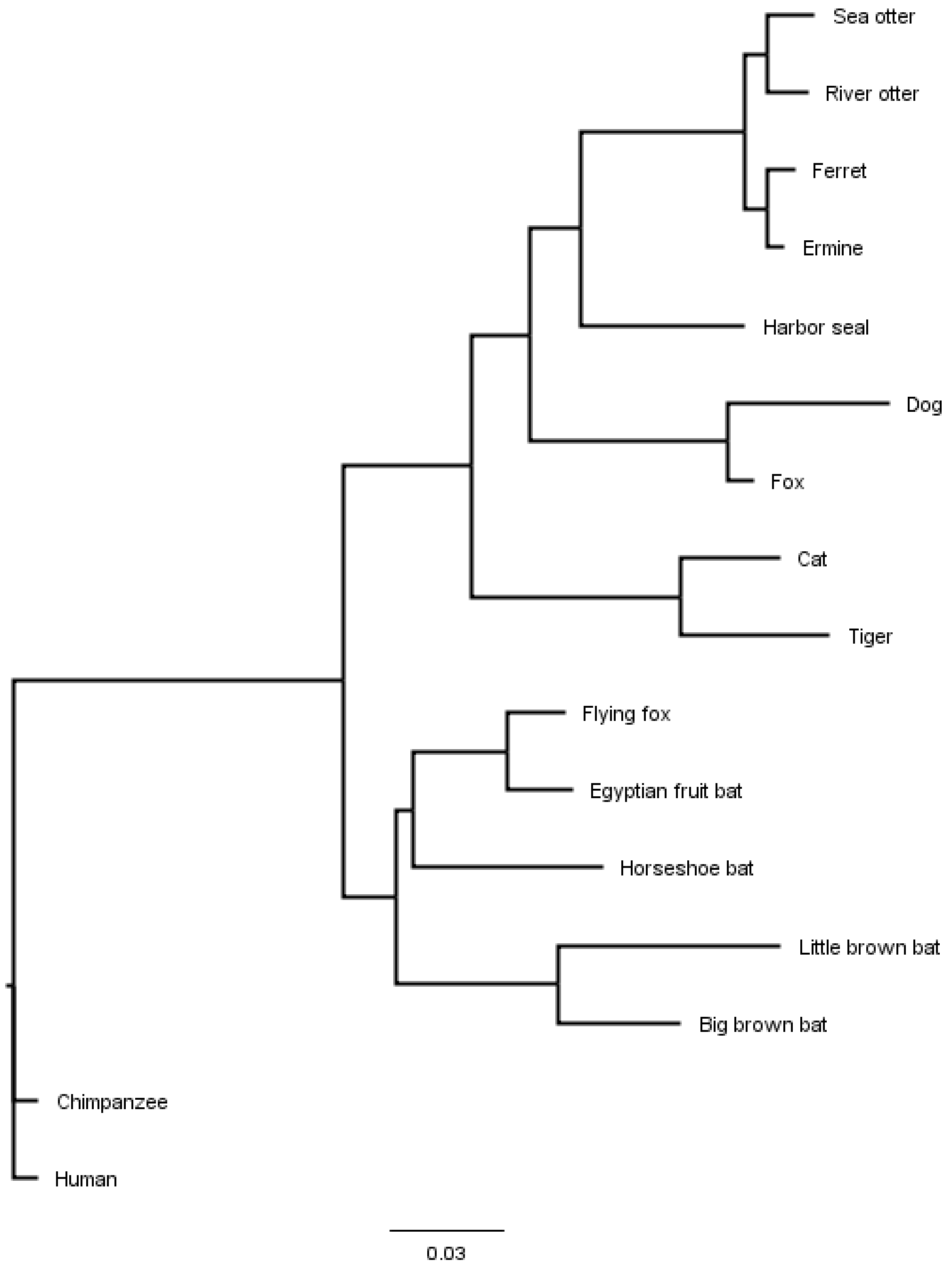

Fig. S5. Maximum likelihood tree of Proprotein Convertase Subtilisin/Kexin Type 5 (PCSK5) among representative mammals. Orthologs were aligned as amino acid sequences (n=16) and a tree generated using RAxML under the JTTγ model with bootstrapping (x=5000) to evaluate reliability of tree topology. Shown in increasing node order. Scale bar corresponds to mutation rate.

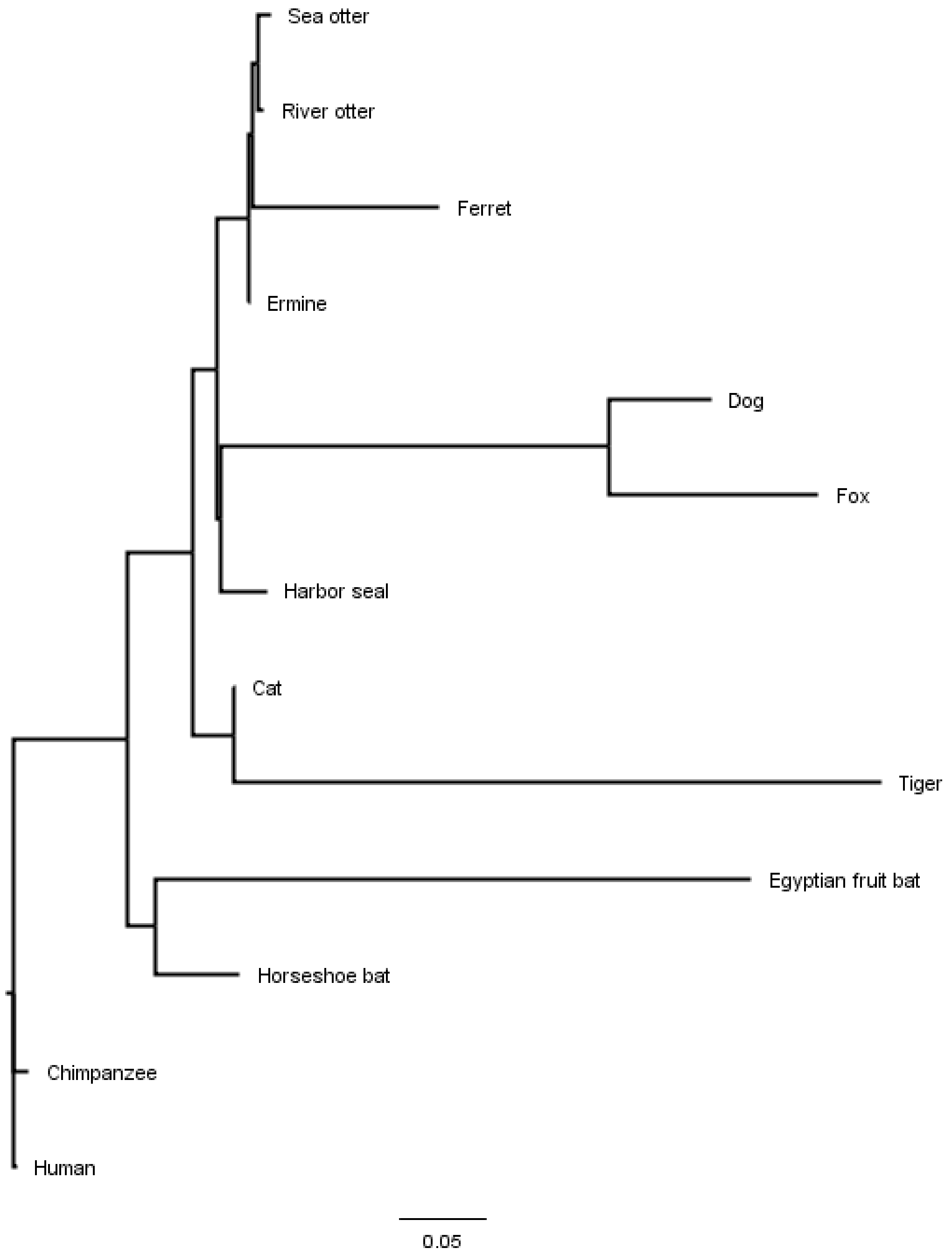

Fig. S6. Maximum likelihood tree of Proprotein Convertase Subtilisin/Kexin Type 6 (PCSK6, PACE4) among representative mammals. Orthologs were aligned as amino acid sequences (n=13) and a tree generated using RAxML under the JTTγ model with bootstrapping (x=5000) to evaluate reliability of tree topology. Shown in increasing node order. Scale bar corresponds to mutation rate.

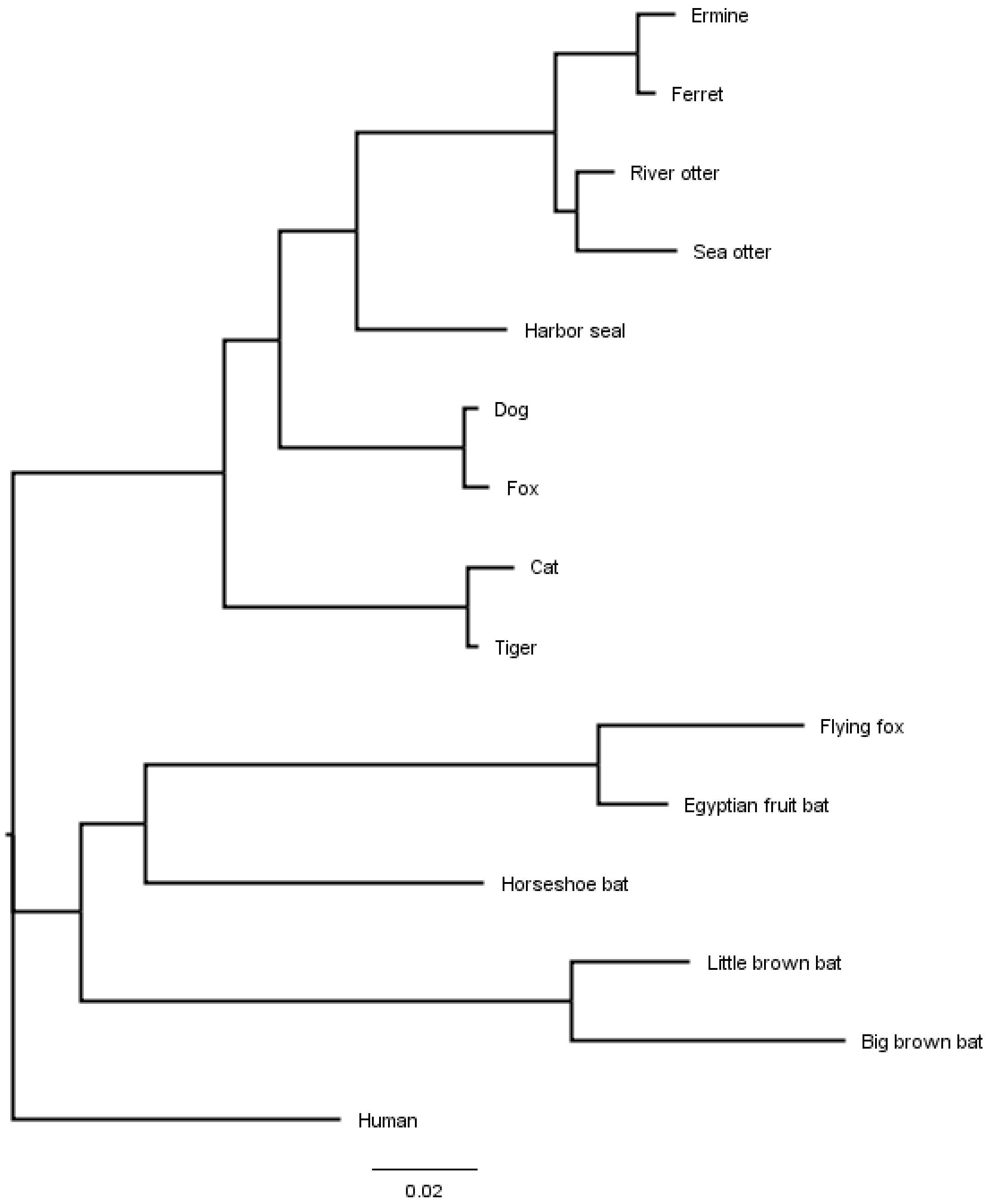

Fig. S7. Maximum likelihood tree of Proprotein Convertase Subtilisin/Kexin Type 7 (PCSK7) among representative mammals. Orthologs were aligned as amino acid sequences (n=15) and a tree generated using RAxML under the JTTγ model with bootstrapping (x=5000) to evaluate reliability of tree topology. Shown in increasing node order. Scale bar corresponds to mutation rate.

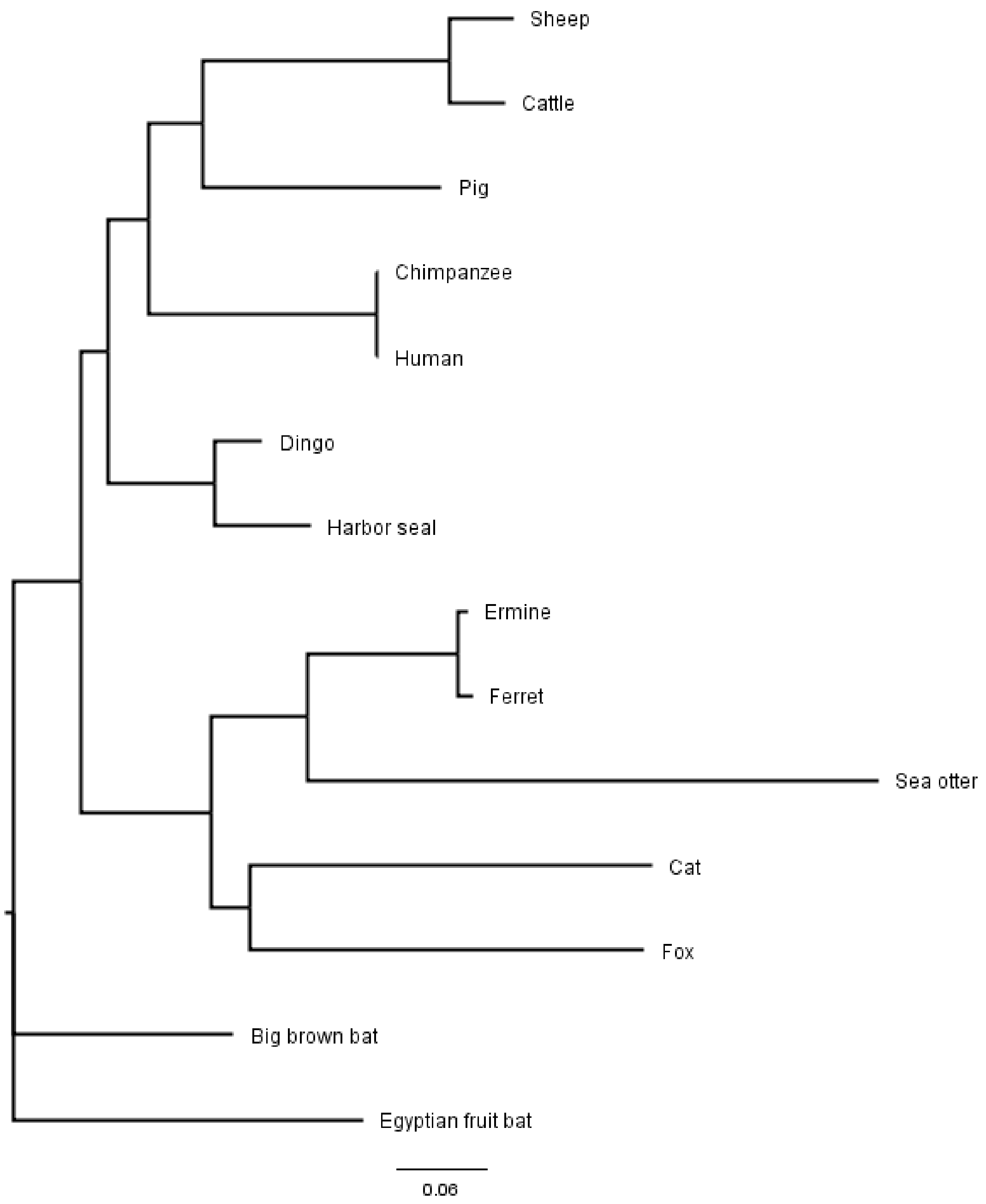

Fig. S8. Maximum likelihood tree of Cathepsin L (CTSL) among representative mammals. Orthologs were aligned as amino acid sequences (n=14) and a tree generated using RAxML under the JTTγ model with bootstrapping (x=5000) to evaluate reliability of tree topology. Shown in increasing node order. Scale bar corresponds to mutation rate.
